# Tomography-Based Investigation on the Carbonation Behavior through the Surface-Opening Cracks of Sliced Paste Specimen

**DOI:** 10.3390/ma13081804

**Published:** 2020-04-11

**Authors:** Dong Cui, Xiaobao Zuo, Keren Zheng, Sudip Talukdar

**Affiliations:** 1 Department of Civil Engineering, School of Science, Nanjing University of Science & Technology, Nanjing 210094, China; Xbzuo1968@163.com; 2 Department of Civil Engineering, Central South University, Changsha 410075, China; zhengkeren@csu.edu.cn; 3 Department of Civil Engineering, British Columbia Institute of Technology, Burnaby, BC 3700, Canada; stalukdar1@bcit.ca

**Keywords:** carbonation, cracking, cement-based materials, attenuation method, computed tomography (CT)

## Abstract

Understanding the cracking behavior during carbonation is of high importance, and the cracks can serve as a shortcut for CO_2_ diffusion, which can further accelerate the carbonation process itself. In this study, a sliced paste sample was taken for an accelerated carbonation test, and the cracking behavior, as well as its impact on carbonation, was investigated through a novel extended attenuation method based on X-ray (XRAM) which is performed primarily on computed tomography (CT). Surface-opening cracks at different carbonation ages were rendered, based on which a full view on the carbonation-cracking behavior was built. The results reveal that the crack paths can rapidly be occupied by CO_2_, and that leads to the generation of V-shaped carbonation cusps pervading the carbonation fronts. The V-shaped carbonation cusps were mostly generated at the early carbonation age (within 14 days), attesting to a less intact sample surface as compared to the inside area. Moreover, this study confirms that the carbonated area would split into two independent zones with variant carbonation degree due to the increased humidity level near the sample surface. The current work reveals the interconnection between carbonation and cracking, and the results can be used for the designing of cement-based materials with better carbonation and cracking resistance.

## 1. Introduction

Carbonation resistance is one of the most-commonly used indexes for the evaluation of concrete durability, and the reaction itself can be simplified as a binding process between the calcium-bearing materials (mainly in the form of portlandite) in concrete and CO_2_ in the atmosphere. The carbonation process gradually consumes the alkali materials in concrete, thus reducing the pH of its pore solution. Next, reduced pH damages the depassivation layer covering the surface of rebar, thereby alluring steel corrosion and causing premature failure of the whole construction [1,2,3].

Since pioneering work by VG Papadakis [4], intensive work regarding concrete carbonation has been carried out [1,3,5,6,7]. Previous research studies revealed that, aside from reduced pH of the pore solution, the carbonation process also refines the microstructure of concrete, thus elevating its mechanical properties [6,8]. Meanwhile, the carbonation process incurs shrinkage, which can further lead to the generation of a honeycomb-like crack network in the carbonated area [9,10,11,12]. Since cracks benefit the invasion of harmful agents like chloride ions [13], sulfate ions [14], and even CO_2_ itself [15], the existence of cracks is detrimental for the survival of the whole construction, and understanding the evolving pattern of carbonation through the surface-opening cracks is therefore of high importance.

Although the necessity of understanding carbonation through cracks has been fully realized, related work is rarely performed due to technical limitations: For traditional testing techniques, for instance, scanning electronic microscopy (SEM), even though the technique is of high resolution, the test requires exposure of the tested area, and the crack morphology may thereby be altered [13,16]; for non-destructive testing (NDT) techniques, for instance, computed tomography (CT), even though exposure of the tested area is no longer necessary, its application on concrete research is still limited due to inadequate resolution [17,18].

CT’s inadequate resolution serves as a critical obstacle for its application in studying the microstructure of cement-based materials, and, in order to mitigate that deficiency, a multiple scanning strategy, which can be regarded as an extension of traditional attenuation method (X-ray attenuation method, XRAM), was developed. In general, extended XRAM is an expansion of the traditional attenuation method, which characterizes physical changes [19], chemical reactions [20,21], and microstructure [20,21] through the difference on attenuation coefficient during variant scans. Moreover, since the investigation of extended XRAM is based on voxel scale, which is commonly of tens of microns, its precision level is much higher than CT [22]. The use of the extended XRAM can be stretched back to the work done by Withjack [23], and later, the method was successfully applied for the investigation of microstructure [24,25], chemical reaction [26,27], and moisture migration [26] for cement-based materials subjected to accelerated carbonation.

In this study, using extended XRAM, both carbonation and cracking behavior were taken for investigation, and the interconnection between carbonation and cracking was built. The use of XRAM for crack identification, to the author’s knowledge, is the first time. The reliability of XRAM in carbonation and crack investigation were examined respectively through thermo-gravimetric analysis (TGA) and through the moments when local carbonation occurred, and, based on the experimental results, a full view on the cracking-carbonation behavior for cement-based material was built. The current work can help attain a deeper understanding on the carbonation mechanism, and the results can be used for designing of cement-based materials with better carbonation and cracking resistance.

## 2. Materials and Methods

### 2.1. Sample Preparation

**Figure 1:**
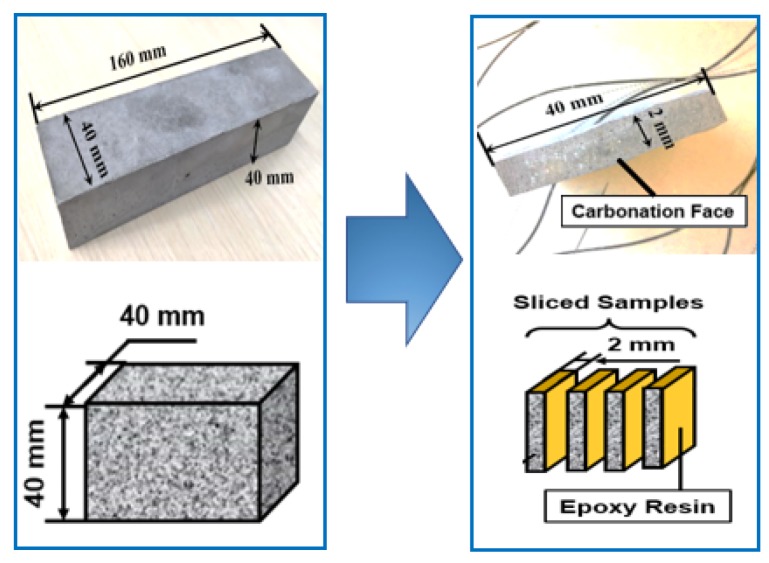
Schematic diagram for sample.

The cement made from the Huaxin cement factory (Huangshi, China) was used in the present study (see Table 1 for its chemical composition). The w/c in the present study was 0.5, under which ratio both carbonation and cracking were easy to occur. Three prisms with the dimensions of 40 × 40 × 160 mm^3^ were cast. All specimens were covered by plastic sheets immediately after casting and were left at ambient condition for 24 h. After that, all specimens were standard-cured (20 ± 1 °C, and humidity level above 95%) for 28 days. Next, a diamond saw was used to cut all specimens into slices (approximately 2 mm per slice; see Figure 1). For each specimen, approximately ten sliced samples were obtained, and one unbroken slice was chosen and was further cured under standard curing condition for another 28 days. The second-time curing was aimed at reducing the damage caused by cutting through self-healing.

**Figure 2:**
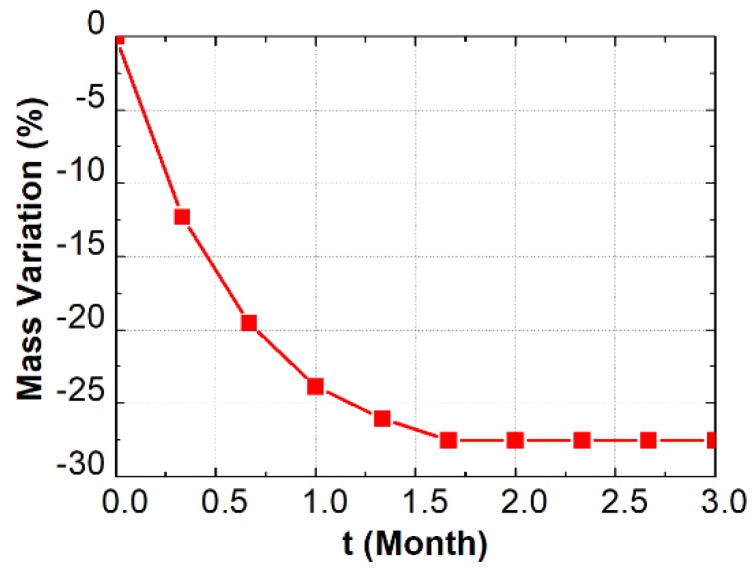
Mass variation of the sample vs. balancing preparation time.

It should be mentioned here that the sliced sample was adopted in this study with the aim of generating a plane crack system. Whereas the plane crack system may deviate from the 3D crack network in bulk specimen, the thin slice makes it easier to reveal the relationship between carbonation and cracking, and the revealed pattern can be reexamined on bulk specimen in the following work. Moreover, a sliced sample can drastically reduce the time required for mass balancing, and a uniform humidity distribution can therefore be achieved in a shorter time (see Figure 2).

### 2.2. Preconditioning and Accelerated Carbonation Test

After curing, the sliced samples were conditioned under constant relative humidity and temperature, through an environmental chamber. The climate inside the chamber was set as 20 ± 1 °C, 45% relative humidity (RH), and nitrogen gas injection was performed periodically to avoid air carbonation. Note that a relative humidity of 45% was selected in this study as the balancing humidity level, which is lower than that based on standard GB T50082-2009 [28], but still within the range of intermediate humidity (45–65%), according to literature [6,29]. The mass of sample was weighed periodically, and its relationship with the drying age was plotted, as shown in Figure 2. The mass curve coincides with that reported elsewhere [30], where the mass of the sample reduced rapidly during the first month, while it tended to be constant after two months of balancing.

After being conditioned in the environmental chamber for three months, four side faces (two 40 × 40 mm^2^, two 40 × 2 mm^2^) were covered by epoxy resin, and the left two faces (40 × 2 mm^2^) were used for 1D accelerated carbonation (see Figure 1). A carbonation chamber was used to carry out the carbonation test, with an inside climate as: 20 ± 3 °C, 65 ± 5% RH, 3% CO_2_ concentration. At 0, 3, 7, 14, and 28 days of carbonation, the sliced samples were taken respectively for experiment.

### 2.3. Testing Methods

#### 2.3.1. Computed Tomography (CT)

Computed tomography (CT) was the primary technique used in this study. In short, CT is capable of obtaining the attenuation coefficient from a local area of the scanned sample, and the data are then reconstructed, forming a series of digital images [31]. As the intensity of the X-ray attenuates as it transmits through the scanned sample, the reduced extent is defined as the attenuation coefficient.

A YXLON CT scanner from Hamburg, Germany, was used here. For all scans, the scanning parameters for CT were held as constant: 0.3 mA current, 195 kV peak energy X-ray, and 3 s of acquisition time per projection. The effective resolution of all CT scans was 63 μm. Note that a relatively higher peak energy of the X-ray and longer acquisition times were selected in the present study, which was aiming at reducing the beam-hardening artifacts.

#### 2.3.2. Extension on Attenuation Method

The extended X-ray attenuation method (XRAM) can be regarded as a combination of traditional attenuation method and X-ray CT. Such a combination is applicable because CT offers the spatial distribution of grayscale value, which indeed was mapped from spatial distribution of the local attenuation coefficient [24]. Therefore, if holding all scanning parameters as constant, physical changes, chemical reactions, and the microstructure can be characterized through altered grayscale values during multiple scans [24,25,26,27].

In this study, XRAM was used to measure the concentration of calcium carbonate ($C\bar{C}$) from local areas. The measurement was made through a dual scan of the sample at two different carbonation ages, and the formed $C\bar{C}$ can thereby be deduced through Equation (1) [27].

(1) $$f_{CaCO_{3}}=\frac{G_{after}-G_{before}}{G_{CaCO_{3}}-\left(1-\frac{38}{369}\right)G_{Ca{\left(OH\right)}_{2}}}$$

where, $G_{before}$ and $G_{after}$ are the grayscale values (GSV) of the voxel at two scanning times, respectively; $G_{CaCO_{3}}$ and $G_{Ca{\left(OH\right)}_{2}}$ are the GSV of calcium carbonate ($C\bar{C}$) and calcium hydroxide (CH), respectively.

The GSV of calcium carbonate and calcium hydroxide were obtained through two additional scans on condensed calcium carbonate and calcium hydroxide powders, respectively, and the 38/369 factor considers the volume change from portlandite to calcite [27]. Moreover, geometrical alignment of CT data from different scan times is necessary before quantitative image analysis, so the registration method developed by Latham et al. [32] was applied here.

#### 2.3.3. Thermo-Gravimetric Analysis (TGA)

Thermo-gravimetric analysis (TGA) was adopted in this study, to examine the reliability of chemical analysis based on XRAM. The specimen was crushed and grounded first, and was then sieved at 70 μm. Next, the powders were freeze-dried, to exclude all evaporable water, and then they were taken for TGA analysis. All TGA tests were based on one Netzch thermal analyzer (Netzch-Gerätebau GmbH, Selb, Germany). The mass of the powders was 30 to 50 mg per test, and the heating procedure ran from 20 to 1000 °C, at 10 °C/min.

## 3. Results

### 3.1. Original CT Data before and after Image Registration

**Figure 3:**
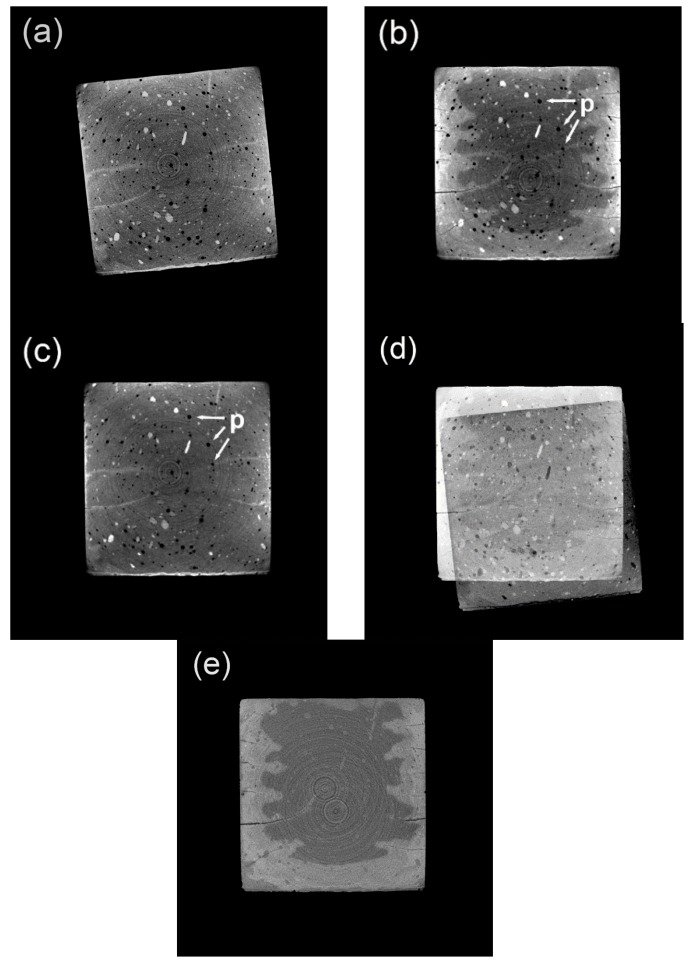
Typical cross-sectional images at different carbonation ages: (**a**) sample at zero days of carbonation (before registration); (**b**) sample at seven days of carbonation; (**c**) sample at 0 day of carbonation (after registration); (**d**) subtracted image through b minus a; and (**e**) subtracted image through b minus c. P, pores.

Figure 3a,b shows the typical CT images rendered from the specimen after zero and seven days of carbonation, respectively, and in Figure 3d, a subtracted image based on Figure 3b minus Figure 3a is presented. Even though the images of Figure 3a,b are of the same slice number, it is evident that the geometrical position of the cross-sectional areas in both figures does not match, and direct image analysis is therefore inapplicable. To mitigate the deficiency, image registration was adopted in this study as an aligning strategy. The registration process was performed on CT data rendered from the specimen after zero days of carbonation, and after image registration, the CT image with a slice number identical to Figure 3b is presented again (see Figure 3c). Compared between Figure 3b,c, noticing that both the geometrical position and the feature points (pores in this case) are satisfyingly matched, the registration progress is proved to be successful. Moreover, judging from the subtracted image (see Figure 3e) based on Figure 3b minus Figure 3c, all the information from the unreacted area is offset, and the remaining bright area illustrates a carbonated area with a clear boundary; thus, denoting it is ready for further image analysis.

### 3.2. Evolved Cross-sectional Areas Based on CT Renderings

**Figure 4:**
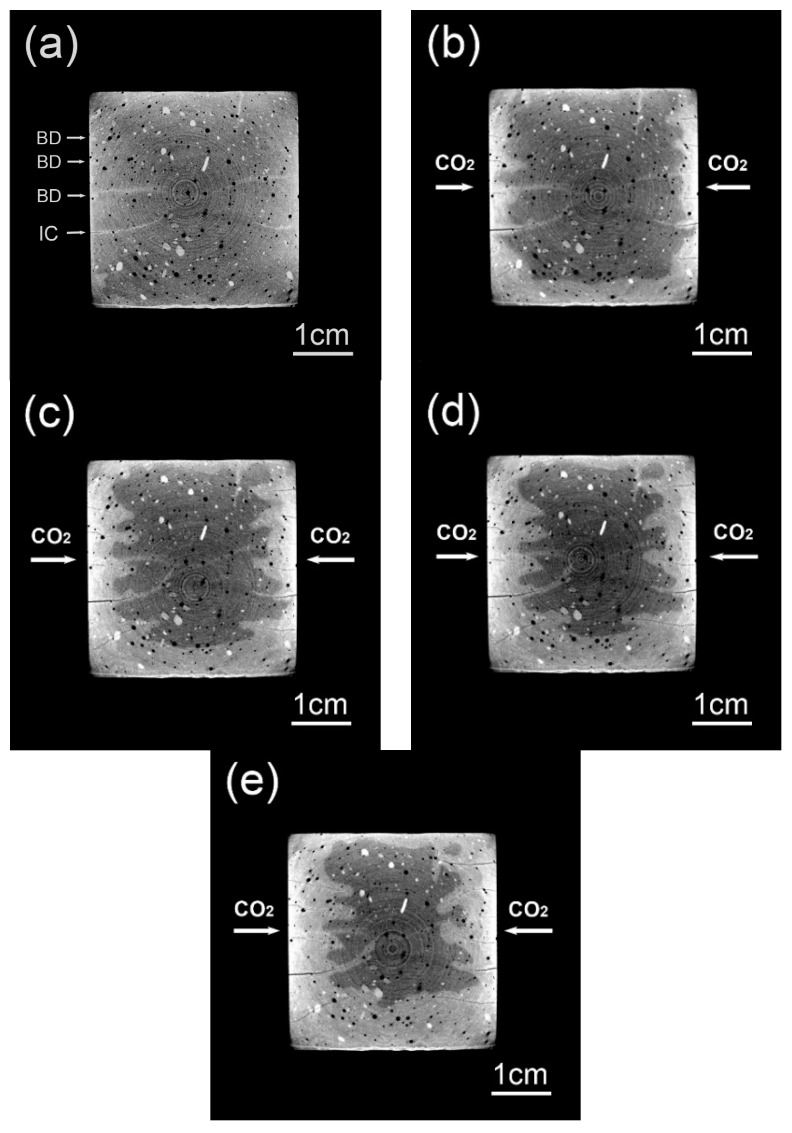
Typical cross-sectional images rendered from specimens after (**a**) 0, (**b**) 3, (**c**) 7, (**d**) 14, and (**e**) 28 days of carbonation, respectively. BD: bright dot caused by natural carbonation; IC: intersected crack.

Figure 4a–e shows typical CT images rendered from the same cross-sectional area after 0, 3, 7, 14, and 28 days of carbonation, respectively. The rendered cross-sectional areas in Figure 4 are strictly matched to each other, which again verifies the reliability of image registration. Compared with the carbonated area, the brightness of the non-carbonated area is darker. The phenomenon was observed intensively before, and a consensus has been attained by now [17,20,26,33]: The carbonation product (mainly calcium carbonate, $C\bar{C}$) possesses a larger molar volume as compared to the reactant (mainly portlandite, CH), and the averaged attenuation coefficient of the non-carbonated area is therefore smaller than that of the carbonated area. Since CT data were mapped from the spatial distribution of local attenuation coefficient, the carbonated area, due to a higher attenuation coefficient, appears brighter. Several ring artifacts due to beam hardening are also visible on Figure 4, but these ring artifacts are mainly restricted to the central part of the images, so their disturbance on carbonation and cracking characterization was limited to later carbonation ages (after 14 days of carbonation). Moreover, please note that a 195 kV was selected as the peak energy of X-ray source, and 3 s was used for the acquisition of each projection, both parameters for CT scans would reduce the beam-hardening artifacts.

The rendered CT images vividly illustrate the evolution of carbonation for the sliced sample: the carbonation process appears more radical within the first 14 days, while being milder at later ages. Moreover, due to the existence of surface-opening cracks, V-shaped carbonation cusps were observed pervading the carbonation fronts. Considering that surface-opening cracks can serve as shortcuts for CO_2_ diffusion, faster early age carbonation speed is reasonable.

According to Figure 4a, at zero days of carbonation, several bright cusps (marked as BD) are already observable. The bright cusps foiled the damaged areas near the sample surface, and these areas seemed to be naturally carbonated already ahead of carbonation test. Moreover, compared between the location for BD in Figure 4a and the location for tapered crack in Figure 4b–e, both locations overlay, thus confirming that surface-opening cracks at later ages were primarily nurtured from the damaged area before carbonation. In addition, Figure 4a also presents several intersected cracks (marked as IC), but their acceleration on carbonation appears insignificant. The insignificant acceleration on carbonation by IC is most likely caused by the blocking of the cracks: The crack openings of the IC had reached the surface of lateral faces before carbonation tests, so during the sealing period (a preconditioning procedure), the crack paths may be blocked by aqueous epoxy resin, and CO_2_ diffusion through the cracks may therefore be hindered. Nevertheless, the blocking effect was restricted to intersected cracks, while for less-propagated cracks, which were under investigation here, the disturbance from epoxy resin sealing is still ignorable.

**Figure 5:**
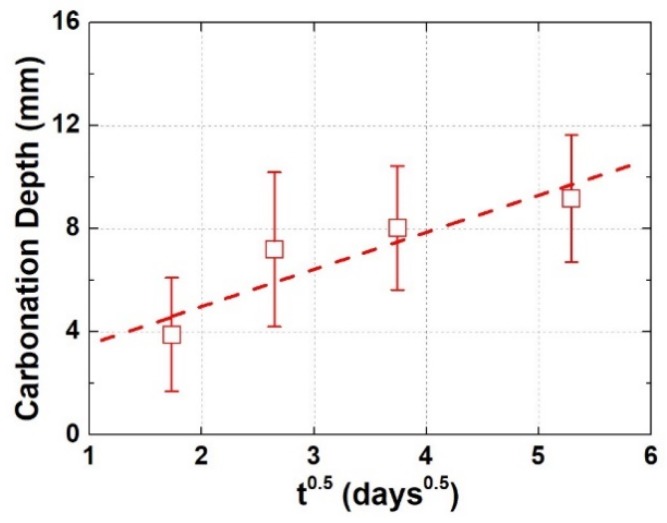
Carbonation depth as a function of the carbonation age.

To quantitatively present the evolution of carbonation extent, carbonation depths at variant carbonation ages were measured based on Figure 4, and the relationship between carbonation depth and square root of carbonation age was then plotted, as shown in Figure 5. The carbonation depth and the square root of carbonation age can still be linearly fitted, suggesting that the carbonation occurred in the present study was mainly a diffusion-controlling progress. The three-day carbonation depth is around 4 cm, which occupies nearly half of the 28-day carbonation depth. Similar with the explanation offered earlier, surface cracking was referred here as the explanation. Besides, at each carbonation age, the carbonation depth displays significant fluctuation (see the error bar in Figure 5), which also reflects the influence of cracking on the carbonation behavior. Moreover, the length of error bar remains nearly stable (approximately 4 mm) throughout the carbonation period, implying that the evolution of cracks and the evolution of carbonation were interconnected with each other.

### 3.3. Spatial Distribution of $C\bar{C}$

**Figure 6:**
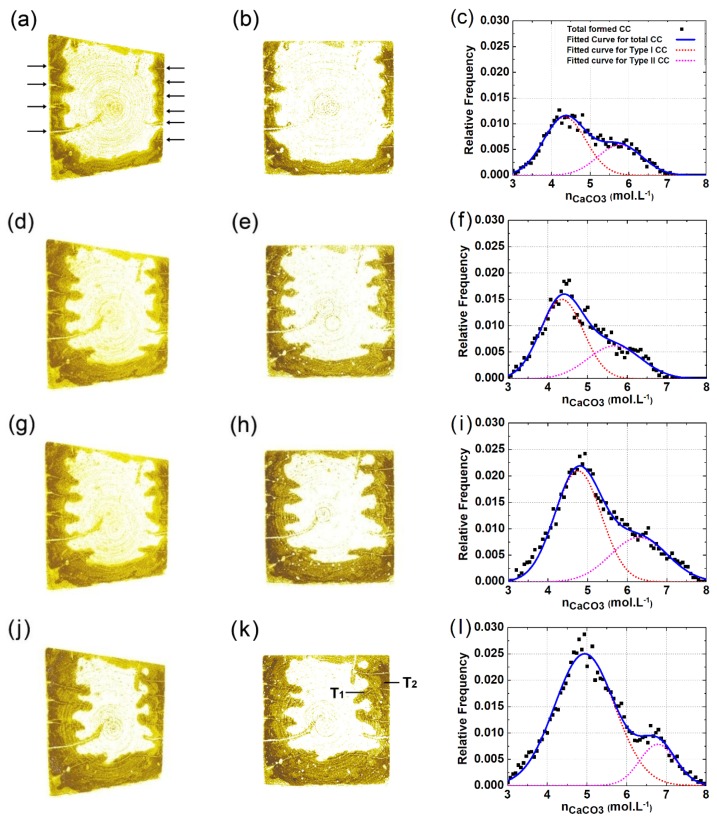
Spatial and frequency distribution of $C\bar{C}$ for sliced sample: (**a**,**d**,**g**,**j**) show the 3D map of $C\bar{C}$ content after 3, 7, 14, and 28 days of carbonation, respectively; (**b**,**e**,**h**,**k**) show the 2D map of $C\bar{C}$ content after, 3, 7, 14, and 28 days of carbonation, respectively; (**c**,**f**,**i**,**l**) show the histograms on local $C\bar{C}$ content based on the related 2D map on its left.

The carbonated area can be isolated through the subtraction of aligned CT slices obtained from different scanning times, as shown in Figure 3. Better yet, if constant scanning parameters were set for all scans, the local $C\bar{C}$ content can be measured, as well, based on the GSV difference from multiple scans, as shown in Equation (1). Figure 6 shows the spatial distribution of local $C\bar{C}$ content for the tested specimen after 3, 7, 14, and 28 days of carbonation, respectively. Compared with Figure 4, the revealed carbonation pattern is similar, but the surface-opening cracks can be more clearly recognized in Figure 6. The increased identifiability of surface-opening cracks is due to natural carbonation: the surface-opening cracks were naturally carbonated ahead of the carbonation test, and several V-shaped carbonation cusps flanking the cracks were therefore formed before carbonation (see BD in Figure 4a); however, in Figure 6, only the $C\bar{C}$ formed between zero days of carbonation, and the test moment were considered, so the cordlike dark tips (pointed with arrows in Figure 6a) contain not only the crack itself, but also the V-shaped carbonation cusp flanking it. Therefore, the dark tips in Figure 6 magnified the actual crack volume, making crack identification easier.

In Figure 6, the cracks march forward at a similar rate with the carbonation front, and that manifests again an interconnection existed between carbonation and cracking. On the one hand, cracks with sufficient width can rapidly be occupied by CO_2_, leading to formation of V-shaped carbonation cusps; on the other hand, the carbonation of crack paths incurs significant shrinkage, which can, in return, “feed” further propagation of cracks.

Local $C\bar{C}$ contents were further analyzed in frequency domain. The work was carried out because varied brightness within the carbonated zone was observed on the $C\bar{C}$ renderings, suggesting different carbonation degree within surface and within V-shaped carbonation cusps flanking inner cracks (see T1 and T2 in Figure 6k). Figure 6c,f,i,l shows the histograms of the local $C\bar{C}$ content at 3, 7, 14, and 28 days of carbonation, respectively. The histogram can be arbitrarily divided into two peaks, and, for convenience, the two types of $C\bar{C}$ are denoted as “Type-I $C\bar{C}$” and “Type-II $C\bar{C}$” hereafter. Compared with Type II $C\bar{C}$, whereas the carbonation degree of Type I $C\bar{C}$ is lower, the percentage of Type I $C\bar{C}$ is significantly larger, and its percentage kept increasing at a faster pace. Redirecting the two types of $C\bar{C}$ on 3D and 2D renderings, it is evident that Type-I $C\bar{C}$ formed mainly at inner cracked areas (for instance, V-shaped carbonation cusps), while the Type-II $C\bar{C}$ formed mainly near the sample surface.

One possible explanation is given here for the different carbonation degree near sample surface and within inner carbonation zones flanking cracks: the tested sample was first balanced under a humidity level of 45%, and it was then carbonated under a humidity level of 65% in this study. Therefore, aside from CO_2_ diffusion, moisture could also migrate from the carbonation chamber into the sliced sample during the carbonation process. The absorption of moisture increased the humidity level of the sample surface, and the high humidity level can gradually increase the local carbonation degree [7,34,35]. Compared with CO_2_ diffusion, the migration rate of moisture was relatively slower, so the carbonated area would gradually split into two zones with variant carbonation degree.

Finally, it was noticed in Figure 6 that the front point of the V-shaped carbonation cusp appears ahead of the crack tip. However, considering that the tip of a tapered crack due to limited width cannot be reached by CO_2_, the crack tip from real specimen should be ahead of the front point for the V-shaped carbonated area [36,37]. The controversy here is still caused by the inadequate resolution of the CT scanner, due to which the crack morphology (especially the tip of the crack) cannot be fully recognized. To mitigate the deficiency, a novel method for crack identification was introduced hereafter.

### 3.4. Method for Detection of Cracks for Partly Carbonated Specimen

**Figure 7:**
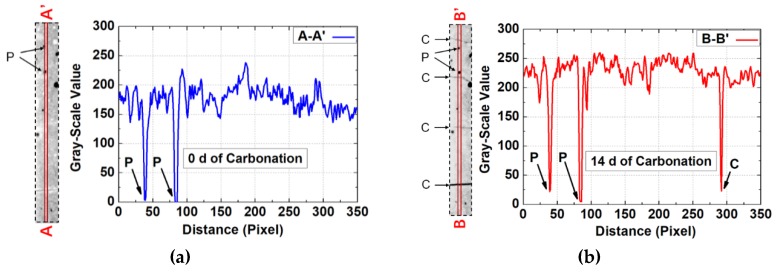

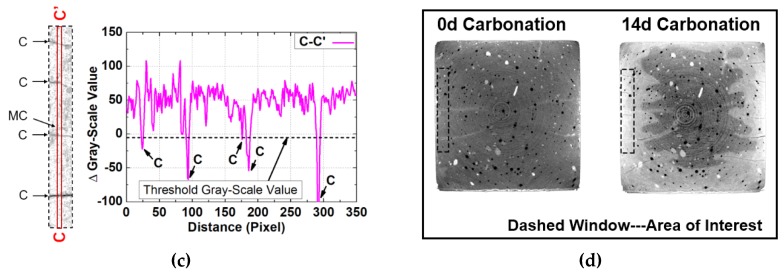
Method for crack detection: (**a**,**b**) represent the identical local areas at 0 and 14 days of carbonation, respectively, and AA’ and BB’ represent 1D grayscale value profiles obtained through linear scanning of the identical area on a and b, respectively; (**c**) represents the local area and 1D profile from (**b**) minus (**a**); and (**d**) represents the cross-sectional area at 0 and 14 days of carbonation, respectively, and the dashed windows represent the local area adopted for investigation.

Figure 7a,b shows magnified local areas of an identical cross-sectional area after 0 and 14 days of carbonation, respectively, and, in each figure, 1D grayscale value profile obtained through linear scanning of the same area is presented (marked respectively as AA’ and BB’). The GSV of BB’ is generally higher than that of AA’, which is reasonable considering that carbonation increased grayscale value. Besides, except macro pores (marked as P) and intersected cracks (marked as C), the location of the cracks cannot be identified directly through 1D grayscale value profile due to the fluctuating nature of grayscale value profiles. In Figure 7c, subtracted image based on Figure 7b minus Figure 7a is presented, and accordingly, the subtraction between BB’ and AA’ is presented as CC’. The CC’ profile reveals mostly positive grayscale values, which is reasonable, as carbonation increases local grayscale value. However, several negative grayscale values are also encountered on the CC’ profile, which foils the trail for cracks.

**Figure 8:**
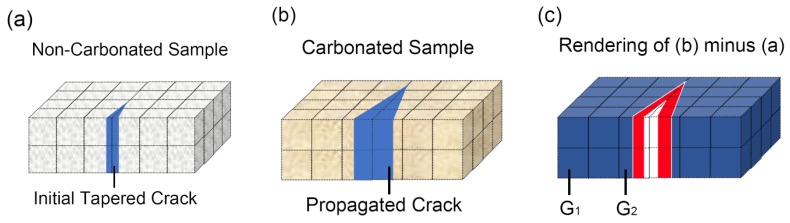
Schematic diagram for the identification of cracks: (**a**) represents typical local area before carbonation; (**b**) represents the identical local area as (**a**) after carbonation; and (**c**) represents the subtracted renderings through (**b**) minus (**a**). The volume of each cubic within the mesh equals the size of one voxel.

To further explain the generation of negative grayscale values on the CC’ profile, a schematic diagram is exhibited in Figure 8. Figure 8a,b presents an identical area before and after carbonation, and the blue V-shaped area in Figure 8a,b presents a surface-opening crack. Obviously, the crack propagated from Figure 8a,b occupied a larger volume. Through a dual CT scan of the tested sample before and after carbonation, the local area in Figure 8a,b can be rendered. Unlike the real sample, the renderings cannot reveal the structure of local areas at scale beneath CT’s resolution. To reflect the deficiency, local areas in Figure 8a,b were meshed, the size of each cubic equals the size of a voxel, and CT tends to be “blind” toward the inner structure within each cubic, as all the information within the cubic has been averaged as one grayscale value (mapped from local attenuation coefficient).

In traditional CT analysis, a threshold grayscale value was carefully selected, and voxels with a grayscale value smaller than the threshold one would be automatically classified as pores or cracks [38,39]. However, the method fails at identifying microcracks, as it is entirely possible that the microcrack only occupies part of a voxel; thus, the grayscale value of the entire voxel still surpasses the threshold grayscale value. In the present study, the subtracted rendering obtained through Figure 8b minus Figure 8a was used for crack identification. For an area containing no cracks or pores, the remaining grayscale value after subtraction was expected to be positive because carbonation increased local grayscale value. For the voxels composed of pores, the remained grayscale value was expected to be 0, as no changes occurred; meanwhile, for the area containing propagated cracks, the local mismatch (marked as red area in Figure 8c) significantly reduces the grayscale value of the investigated voxel. If this negative effect surpasses the positive effect caused by carbonation, a negative grayscale value would occur on the subtracted renderings. Note that although noises from CT data also introduce a negative grayscale value, their disturbance on crack identification can be mitigated, as the noise-induced crack does not acquire a cordlike crack geometry; thus, it can be filtered through artificial reexamination.

Redirecting to Figure 7c, based on the negative grayscale values on the CC’ profile, the surface-opening cracks can be detected. Note that one micro crack, which cannot be discerned on Figure 7b, was observed, and the efficiency of current method in crack identification was revealed.

### 3.5. Rendered Cracking Behavior during Accelerated Carbonation

**Figure 9:**
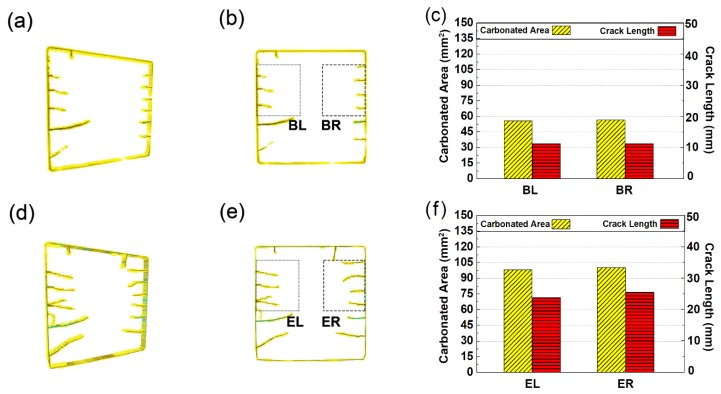

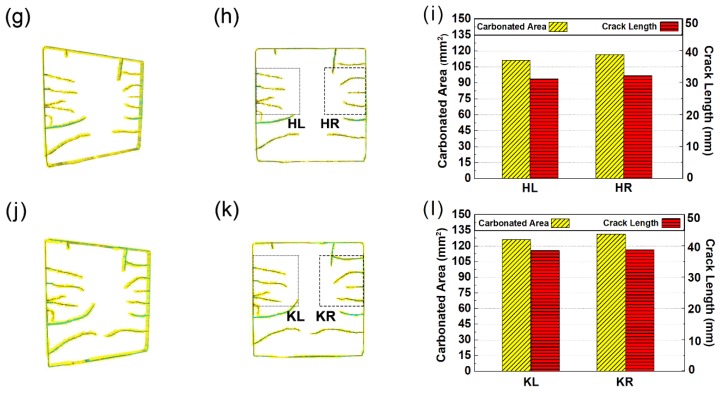
Spatial renderings for the propagated cracks: (**a**,**d**,**g**,**j**) show the 3D map of propagated cracks after 3, 7, 14, and 28 days of carbonation, respectively; (**b**,**e**,**h**,**k**) show the 2D map of propagated cracks formed after 3, 7, 14, and 28 days, respectively; (**c**,**f**,**i**,**l**) show the carbonated area and total crack length based on the related 2D map on its left.

Based on the method offered above, the spatial distribution of cracks is rendered, as shown in Figure 9. The rendered cracks in Figure 9 appear close to that in Figure 6, thus revealing its reliability. Moreover, considering that mismatch caused by crack propagation is used here for crack identification, the revealed crack morphology in Figure 9 can serve as compelling evidence for the propagation of cracks.

Figure 9 reveals that, similar to the evolution of carbonation, the cracks propagated at a faster speed within the first 14 days, but at a slower speed within later ages. To quantitatively illustrate the relationship between cracking and carbonation, the total propagated crack length and the total volume percentage of the carbonated area were measured, respectively. The measurement was made within two rectangular areas (see the dashed and dotted rectangular windows in Figure 9) for each carbonation age, and the result is shown in Figure 9c,f,i,l, respectively.

As shown on all bar graphs in Figure 9, at each carbonation age, the total crack length and the carbonated area in the left and right rectangular windows are similar, suggesting that the window size is sufficient. In particular, at three days of carbonation, even though the distance between adjacent cracks is larger in the left window (see Figure 9b), due to faster propagating speed, the total crack lengths within both rectangular windows are still similar. Combining the fact that carbonated areas in the left and right windows are close, the similar crack length here, again, suggests an interaction existed between crack propagation and carbonation.

## 4. Verifications

In this study, the cracking behavior of cement-based material subjected to accelerated carbonation was rendered through a novel crack-detecting method. Moreover, based on the spatial distribution of $C\bar{C}$, the carbonation degrees for the sample surface and for the inner area flanking the surface-opening cracks were revealed to be different. To examine the reliability of the experimental results, verification work was performed hereafter.

### 4.1. Verification Based on the Spatial Distribution of $C\bar{C}$ Formed during Each Reaction Interval

As described earlier, tracing the behavior of cracks remains a hard task at the current stage, and it is therefore difficult to verify the surface-opening cracks revealed from this study. Luckily, consider that cracks can serve as shortcuts for CO_2_ diffusion, leading to the formation of V-shaped carbonation cusps flanking the tapered cracks, the propagation of cracks can still be indirectly reexamined based on the moments when new V-shaped carbonation cusps were formed.

**Figure 10:**
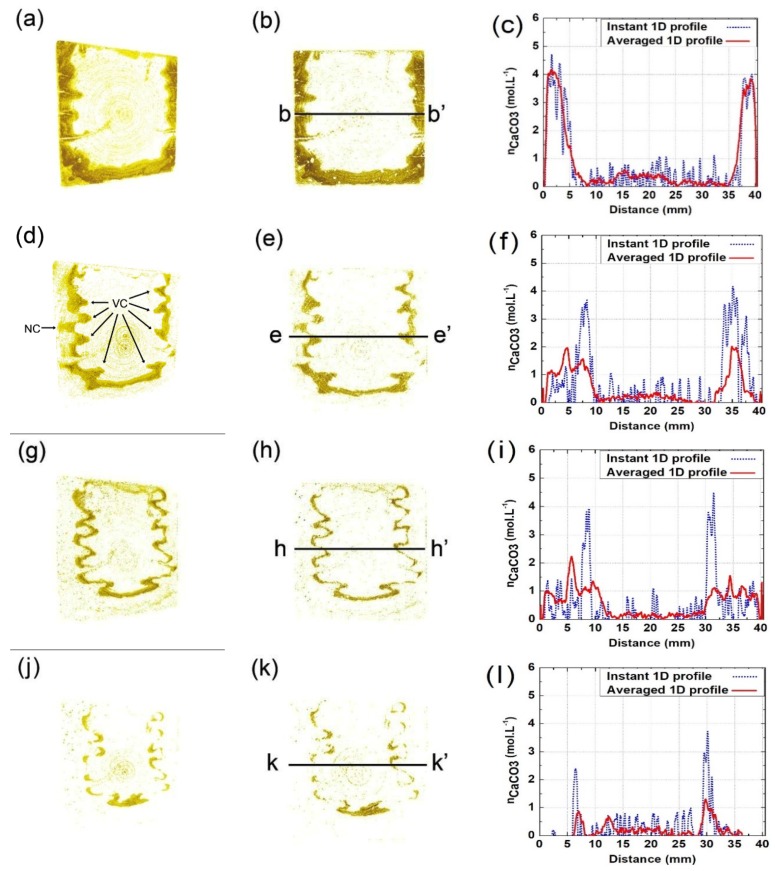
Spatial distributions of $C\bar{C}$ content: (**a**,**d**,**g**,**j**) show the 3D map of $C\bar{C}$ content formed between 0 and 3 days; 3 and 7 days; 7 and 14 days; and 14 and 28 days, respectively. (**b**,**e**,**h**,**k**) Show the 2D map of $C\bar{C}$ content formed between 0 and 3 days; 3 and 7 days; 7 and 14 days; and 14 and 28 days, respectively. (**c**,**f**,**i**,**l**) show the 1D $C\bar{C}$ profile along b–b’, e–e’, h–h’, and k–k’ and averaged 1D profiles shown in (**b**,**e**,**h**,**k**), respectively.

Figure 10 shows the spatial distribution of newly formed $C\bar{C}$ between each time interval (0 and 3 days; 3 and 7 days; 7 and 14 days; and 14 and 28 days). The emergence of V-shaped carbonation areas is more frequently shown within the first 14 days, which echoes the drastic propagation of cracks during the early carbonation age in Figure 9. Besides, the emergence of newly generated V-shaped cusps shows high consistency with the propagation of surface-opening cracks revealed in Figure 9, which verifies the reliability of the crack-detecting method. A case in point is shown in Figure 10d, where a newly carbonated area (marked as NC) was formed. The emergence of NC seemed to be quite delayed, as the area was quite close to the carbonation face. However, the formation of NC coincided with the newly-propagated micro-crack (marked as MC in Figure 7c) revealed in this study; therefore, it validates the reliability of the current crack-detecting strategy.

In order to verify the varied carbonation product near the sample surface and within V-shaped carbonation area flanking cracks, 1D distributions of $C\bar{C}$ (instant $C\bar{C}$ profiles) were further measured through linear scanning of the related areas on its 2D renderings, and the results are shown in Figure 10c,f,i,l, respectively. As shown on these 1D profiles, even though the surface areas have been carbonated already within the early carbonation age, newly formed $C\bar{C}$ is still detectable near the sample surface during the later age, and that explains the increase on the carbonation degree near the sample surface, in other words, formation of the Type-II $C\bar{C}$ area.

### 4.2. Verification Based on Thermo-Gravimetric Analysis (TGA)

**Figure 11:**
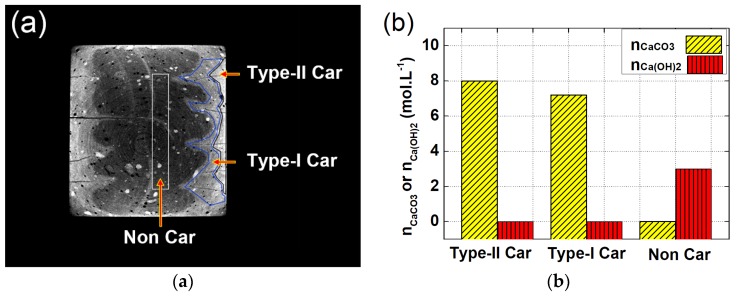
Sampled areas and experimental results for TGA: (**a**) selection of the sampling areas based on rendered CT slice; (**b**) measured CH and $C\bar{C}$ concentration from variant local areas.

Thermo-gravimetric analysis (TGA) was adopted to verify the existence of carbonation gradient along the carbonation depth. To fulfill that, inner non-carbonated area, local carbonated area near sample surface, and local carbonated area flanking the cracks were sampled, respectively, for TGA tests. The sampled areas can be precisely determined with the assistance of CT images, as shown in Figure 11a.

Figure 11b shows the CH and $C\bar{C}$ concentrations measured from all three local areas. Consistent with former description, the inner carbonated area flanking the cracks was denoted as “Type-I Car”, the outer carbonated surface was denoted as “Type-II Car”, and the inner non-carbonated area was denoted as “Non Car”. Compared with “Type-I Car”, the carbonation degree of “Type-II Car” was elevated, as the $C\bar{C}$ concentration of “Type-II Car” was higher. Besides, in view of the fact that calcium hydroxide (CH) was depleted in both Type-I Car and Type-II Car, the increase on carbonation degree for Type-II Car was mainly attributed to the carbonation of calcium bearing phases other than calcium hydroxide (CH).

As a conclusion, through investigations on the moments when local carbonation occurred and on the chemical compositions from variant local areas, the reliability of the experimental results in this study was verified, and a full view on the carbonation-cracking behavior can therefore be offered.

## 5. A Full View on the Carbonation-Cracking Behavior

Based on the propagating behavior of cracks, and considering that evolved pattern of moisture during carbonation, a full view of carbonation and cracking behavior was built.

**Figure 12:**
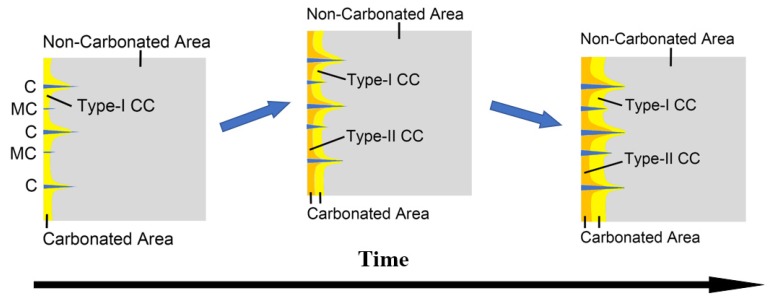
Full view of carbonation process for a cracked paste slice.

Figure 12 presents the schematic for the cracking behavior of a sliced paste specimen during carbonation, and the whole progress can be divided into three stages. The initial stage describes the early age cracking behavior, during which period the CO_2_ rapidly occupies the surface-opening cracks, forming an irregular-shaped carbonation front with V-shaped carbonation cusps flanking the tapered cracks (see the first figure in Figure 12). Although the initial stage only lasts for a short period (less than three days in this study), significant carbonation shrinkage accumulates during this period, and that stress serves as an energy source, feeding the later propagation of micro cracks.

At the middle stage (see the second figure in Figure 12), with the marching forward of the carbonation front, the existed micro cracks at the initial stage, after gaining sufficient propagation, can also serve as shortcuts for CO_2_ diffusion, leading to generation of new V-shaped carbonation cusps. Meanwhile, migration of moisture, which acquires a slower rate as compared to migration of CO_2_, gradually increases the humidity level near the sample surface, and the carbonated area starts to split into two independent zones: one carbonation area with a higher carbonation degree near the surface (type-II $C\bar{C}$), and one carbonated area with a lower carbonation degree around the core area (type-I $C\bar{C}$).

At the late carbonation stage (see the last figure in Figure 12), as the carbonation front has reached the inner area of the specimen, where the microstructure is relatively more intact as compared to that near the sample surface, due to limited micro cracks, no new V-shaped carbonation cusps are generated, and both carbonation and cracking march forward at a slower rate, as compared to the former two stages.

## 6. Conclusions and Further Work

### 6.1. Conclusions

With the assistance of an extended attenuation method (XRAM), a novel method on crack detection was introduced. Based on the new method and computed tomography (CT), the carbonation-cracking behavior of the paste specimen subjected to accelerated carbonation was investigated, and a full view on the carbonation-cracking behavior was built. Several conclusions can be drawn from the present study.

(1) Compared with raw CT data, the identifiability of cracks was higher on the $C\bar{C}$ renderings obtained through XRAM, as the dark cordlike strips on the $C\bar{C}$ renderings were composed of both the crack itself and the naturally carbonated area flanking it.
(2) Carbonation incurs significant propagation of surface-opening cracks, due to which the grayscale value of local areas may decrease, and the decreased grayscale value can therefore be used as a clue for the identification of cracking.
(3) Surface-opening cracks can serve as shortcuts for CO_2_ diffusion, leading to the formation of V-shaped carbonation cusps pervading the carbonation front and faster early age carbonation speed; in return, the shrinkage accumulated during carbonation can serve as the energy source, feeding further propagation of the surface-opening cracks.
(4) Due to variant migrating rates of moisture and CO_2_, the carbonated area would divide into two independent zones with variant carbonation degrees. The carbonation degree for the sample surface was higher, while the carbonation degree for the inner carbonated zone flanking cracks was relatively lower.
(5) Based on the carbonation-cracking behavior, the carbonation process can be divided mainly into three stages. At the first and second stages, cracks were propagating rapidly, accompanying drastic formation of V-shaped carbonation cusps, while at the third stage, due to finer microstructure at inner areas, both the carbonation and the cracking speed were slower.

### 6.2. Future Work

In this study, XRAM was applied for the first time for the crack identification, and the carbonation-cracking behavior for paste slices was thus revealed. Nevertheless, current work was based on paste specimens made of ordinary cement, and the influence of supplementary materials on carbonation was therefore not taken into consideration. In order to guarantee a systematic work, specimens blended with supplementary materials should also be taken for investigation. Moreover, considering that aggregate can entangle cracks, which will alter the cracking behavior, mortar and concrete specimens should be investigated in the future. Through comparing the carbonation-cracking patterns for cement-based materials with variant compositions, concrete with finer cracking and carbonation resistance can be designed.

Moreover, as a universal crack-identification method, the strategy used for crack characterization should also work for cement-based materials subjected to other durability issues, like calcium leaching, sulfate attack, chloride penetration, and so on, and the interconnection between cracking and these durability issues can, therefore, also be investigation through similar strategy. These related works continue in our laboratory.

## Figures and Tables

**Table 1:** Cement composition from this study (%).

Cement	CaO	SiO_2_	Al_2_O_3_	Fe_2_O_3_	MgO	SO_3_	Others	LOI	Total
Mass ratio	62.60	21.35	4.67	3.31	3.08	2.25	1.29	1.45	100
